# Antibacterial and Antifungal Activity of Zinc(II) Carboxylates With/Without N-Donor Organic Ligands

**DOI:** 10.1155/MBD.2002.269

**Published:** 2002

**Authors:** V. Zelenák, K. Györyová, D. Mlynarcík

**Affiliations:** 1 Department of Inorganic Chemistry P. J. Safárik University Moyzesova 11 Kosice 041 54 Slovakia; 2 Faculty of Pharmacy Comenius University Odbojárov 10 Bratislava 832 32 Slovakia

## Abstract

The antibacterial and antifungal activity of zinc(II) carboxylates with composition Zn(RCOO)_2_•nH_2_O(R =H-, CH_3_^-^, CH_3_CH_2_CH_2_^-^, (CH_3_)_2_CH-, XCH_2_^-^, X=Cl, Br, I, n=0 or 2), [ZnX_2_(Nia^+^CH_2_COO^-^)_2_](Nia=nicotinamide, X=Cl, Br, I) and [Zn(XCH_2_COO)_2_(Caf)_2_]•2H_2_O (Car=caffeine, X=Cl, Br) is studied against bacterial strains *Staphylococcus aureus*, *Escherichia coli* and yeast *Candida albicans*. The structural types are assigned to the prepared compounds and the influence of (*i*) carboxylate chain length, (*ii*) substitution of hydrogen atom of carboxylate by halogen and (*iii*) presence of N-donor organic ligands on the biological activity is discussed.


### ANTIBACTERIAL AND ANTIFUNGAL ACTIVITY OF ZINC(II) CARBOXYLATES WITH/WITHOUT N-DONOR ORGANIC LIGANDS

V. Zelenák*[1], K. Györyová[1] and D. Mlynarcík[2]

[1] Department of Inorganic Chemistry, P. J. Safárik University, Moyzesova 11, 041 54 Kosice, Slovakia zelenak@kosice.upjs.sk

[2] Faculty of Pharmacy, Comenius University, Odbojárov 10, 832 32 Bratislava, Slovakia

### ABSTRACT

The antibacterial and antifungal activity of zinc(II) carboxylates with composition $Zn(RCOO)_2 \cdot nH_2O$ (R =H-, $CH_3$-, $CH_3CH_2CH_2$-, $(CH_3)_2CH$-, $XCH_2$-, X=Cl, Br, I, n=0 or 2), $[ZnX_2(Nia^+CH_2COO^-)_2]$ (Nia=nicotinamide, X=Cl, Br, I) and $[Zn(XCH_2COO)_2(Caf)_2] \cdot 2H_2O$ (Caf=caffeine, X=Cl, Br) is studied against bacterial strains *Staphylococcus aureus, Escherichia coli* and yeast *Candida albicans*. The structural types are assigned to the prepared compounds and the influence of (*i*) carboxylate chain length, (*ii*) substitution of hydrogen atom of carboxylate by halogen and (*iii*) presence of N-donor organic ligands on the biological activity is discussed.

### INTRODUCTION

Zinc is known to have a considerable effect on many biological processes. This element has structural and gene-regulatory functions in living organisms and participates in catalysis of essential metabolic reactions. It is known to regulate activity over 300 metalloenzymes [1, 2]. Zinc is a component of special proteins, called "zinc fingers", which recognize DNA base sequence and thus serve in the selective activation and regulatory control of gene transcription, i.e. zinc participates in the reliable transfer of genetic information [3].

The wound-healing effect of zinc-containing ointments is known for several centuries [3]. The zinc may be used as a therapeutic agent and it may act as an antisickling agent and play role in the prevention of pain crisis in sickle-cell disease. Zinc was successfully used in the treatment of acrodermatitis enteropathica, Wilson's disease, gastrointestinal disorders, infertility and other diseases [4]. Very important is antibacterial and antiviral effect of the zinc(II) ion [5, 6].

Zinc(II) complexes with organic molecules were also used in clinical medicine, e.g. complex of zinc(II) acetate with erythromycin is used for acne therapy [7]. In general, organic ligands can contribute to better transport of metal ions through the lipophillic regions of cell membranes. However, it is also possible that some metal complexes are not able to reach their site of action in sufficient concentration due to their decreased solubility [8]. For the preparation of effective antimicrobial species it is very important to gain knowledge about the structure and bonding relations in the compounds. It was found that the antibacterial effect of various drugs could be enhanced when they are chelated to metal [9].

In order to understand properties of the zinc we have synthesized zinc(II) complexes with/without N-donor organic ligands (nicotinamide, caffeine). Their structure and thermal properties have been described elsewhere [10-17]. The present work deals with the study of the antibacterial and antifungal properties of these reported compounds.

### MATERIALS AND METHODS

The syntheses of the complexes have been described elsewhere [10-17]. All chemicals used in the syntheses were analytical grade and purchased from Aldrich Chemical Company.

A Specord M80 Infrared Spectrophotometer was used for infrared (IR) spectroscopic analyses. IR spectra were obtained as KBr discs.

The compounds were screened for their antibacterial activity against strains *Staphylococcus aureus Mau 29/58*, *Escherichia coli Ec 377/79* and *Candida albicans 45/53*.The strains originated from the former Czechoslovak Type Culture Collection in Prague. The compounds were tested in diluted culture media (nutrient broth for bacteria, Sabouraud's medium for fungi). Serial dilutions of the compounds under study were prepared in the culture media and 24 hr culture of microorganisms was added to each dilution. After 24 h cultivation (bacteria 37 °C, fungi 25 °C) minimum inhibitory concentration (MIC) [μg/ml] was determined for each compound as the lowest concentration which was capable of stopping the microbial growth. For evaluation of the biological activity the compounds with the MIC upto 100 μg/ml were considered as very good active, compounds with MIC in the range 100 - 1000 μg/ml were considered to possess good biological activity, compounds with MIC in the range 1000 - 5000 μg/ml weak, and if MIC is above 5000 μg/ml the compound were taken as biologically inactive.

### RESULTS AND DISCUSSION

On the basis of the results of X-ray diffraction and/or IR spectra published elsewhere (see Table 1), the structural types were assigned to the studied compounds (see Fig. 1 and Table 1). In case only IR spectral data were available, the assignment was based on:

(i) comparisons of the IR spectra made within a series of closely related compounds, several of which have X-ray structural data available

(ii) the fact that for unidentate coordination of carboxylate group inequality both of C-O bonds is characteristic and therefore a larger value of carboxylate separation (>170 $cm^{-1}$), defined as $\Delta = \nu_{as}(COO^-) - \nu_s(COO^-)$, is observed in comparison to bidentate mode of coordination

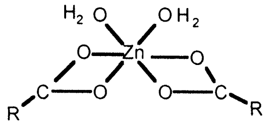

A

Compound II

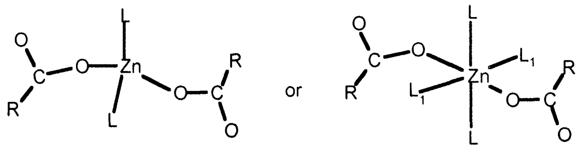

L, L1 = water or organic ligand

B

Compounds III, IV, V, VI, XI, XII

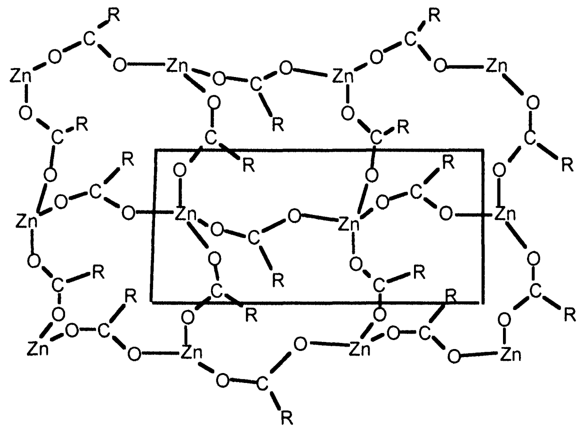

C

Compounds VII, XIII, XIV

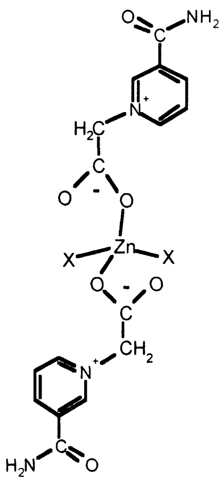

X = Cl $^-$, Br $^-$, I $^-$

D

Compounds VIII, IX, X

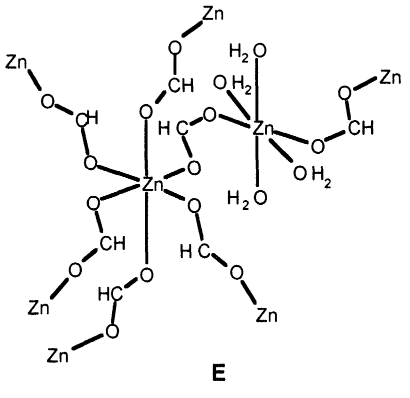

E

Compound I

Fig. 1: The structural types assigned to the prepared compounds

For example, in zinc acetate dihydrate (**II**), the value of separation of the carboxylate stretches, $\Delta$=108 $cm^{-1}$ [19]. This value characterizes the bidentate mode of carboxylate coordination and it is in agreement with the results of X-ray diffraction [20]. In the substituted analogues $Zn(ClCH_2COO)_2 \cdot 2H_2O$ (**V**) and $Zn(BrCH_2COO)_2 \cdot 2H_2O$ (**VI**) the $\Delta$ values (196 $cm^{-1}$) were rather higher in comparison to $Zn(CH_3COO)_2 \cdot 2H_2O$ (see Table 1), indicating monodentate carboxylate coordination and molecular structure of the compound.

On the contrary to the chloro- and bromo- derivatives, in zinc iodoacetate $Zn(ICH_2COO)_2$ (**VII**), the value of separation of carboxylate stretches, $\Delta$=144 $cm^{-1}$, characterizes the bidentate mode of carboxylate coordination. Moreover, splitting of both carboxylate stretches observed in the sample, indicated the presence of carboxylate bridges (*syn-syn or syn-anti*) [21]. Therefore, two-dimensional structure was assigned to the zinc(II) iodoacetate, similarly to the structure of anhydrous zinc acetate $Zn(CH_3COO)_2$, where *syn-anti* bridges are present [22]. The same method was applied to the assignment of the structural types to the other prepared compounds. The values of separation of $-CO_2$ stretches and assigned structural types are summarized in Table 1 and shown in Fig. 1.

Table 1. The values of the separation of the $-CO_2$ stretches and assignment of the structural types to the studied compounds

| Compound | $\Delta=\nu_{as}(COO^-)-\nu_s(COO^-)$ [$cm^{-1}$] | Structural type *;** | Ref. |
|---|---|---|---|
| $Zn(HCOO)_2 \cdot 2H_2O$ **(I)** | 152 | 3D * | 23 |
| $Zn(CH_3COO)_2 \cdot 2H_2O$ **(II)** | 108 | M * | 19, 20 |
| $[Zn(CH_3COO)_2(Nia)_2] \cdot 2H_2O$ **(III)** | 172 | M ** | 18 |
| $[Zn(CH_3COO)_2(Caf)_2] \cdot 2H_2O$ **(IV)** | 170 | M ** | 10 |
| $Zn(ClCH_2COO)_2 \cdot 2H_2O$ **(V)** | 196 | M ** | 13 |
| $Zn(BrCH_2COO)_2 \cdot 2H_2O$ **(VI)** | 196 | M ** | 18 |
| $Zn(ICH_2COO)_2$ **(VII)** | 144 | 2D ** | 18, 22 |
| $[ZnCl_2(Nia^+CH_2COO^-)_2]$ **(VIII)** | 276 | M * | 15, 16 |
| $[ZnBr_2(Nia^+CH_2COO^-)_2]$ **(IX)** | 276 | M * | 11, 16 |
| $[ZnI_2(Nia^+CH_2COO^-)_2]$ **(X)** | 280 | M * | 14, 16 |
| $[Zn(ClH_2COO)_2(Caf)_2] \cdot 2H_2O$ **(XI)** | 200 | M ** | 13 |
| $[Zn(BrCH_2COO)_2(Caf)_2] \cdot 2H_2O$ **(XII)** | 188 | M * | 18 |
| $Zn(CH_3CH_2CH_2COO)_2$ **(XIII)** | 130 | 2D * | 24 |
| $Zn((CH_3)_2CHCOO)_2$ **(XIV)** | 140 | 2D ** | 17 |

Abbreviations: * - structure assigned on the basis of spectral data and X-ray diffraction
** - structure assigned on the basis of spectral data
M - molecular structure
2D - two-dimensional structure
3D - three-dimensional structure

*Antimicrobial activity*

The results of testing of antibacterial and antifungal activity of the prepared compounds against bacterial strains *Staphylococcus aureus*, *Escherichia coli* and yeast *Candida albicans* are summarized in Table 2.

From the Table 2 it can be seen, that the zinc(II) carboxylates have the highest efficiency on the Gram-positive bacteria *Staphylococcus aureus*. The efficiency on the Gram-negative bacteria *Escherichia coli* is lower and that on the fungi *Candida albicans* is negligible.

The influence of the carboxylate chain length on the biological activity is shown in Fig. 2. The increasing of the carboxylate chain length may affect the lipophillicity of the molecule. In general, as the chain length increases lipophillicity of the compound increases and its solubility in the water decreases. From the Fig. 2 it is obvious that the length of the carboxylate chain affects the activity of the zinc(II) carboxylates neither against the *Staphylococcus aureus*, nor *Escherichia coli*. This is probably due to low thermodynamic stability of the compounds and their dissociation to ions in the solution.

Table 2. Antimicrobial activity of the studied compounds (MIC in μg/ml)

| Compound | S. aureus | E. coli | C. albicans |
|---|---|---|---|
| $Zn(HCOO)_2 \cdot 2H_2O$ **(I)** | 312 | 5000 | 625 |
| $Zn(CH_3COO)_2 \cdot 2H_2O$ **(II)** | 312 | 5000 | 1250 |
| $[Zn(CH_3COO)_2(Nia)_2] \cdot 2H_2O$ **(III)** | 1250 | 5000 | 5000 |
| $[Zn(CH_3COO)_2(Caf)_2] \cdot 2H_2O$ **(IV)** | 5000 | >5000 | >5000 |
| $Zn(ClCH_2COO)_2 \cdot 2H_2O$ **(V)** | 312 | 2500 | 1250 |
| $Zn(BrCH_2COO)_2 \cdot 2H_2O$ **(VI)** | 156 | 312 | 1250 |
| $Zn(ICH_2COO)_2$ **(VII)** | 78 | 312 | 1250 |
| $[ZnCl_2(Nia^+CH_2COO^-)_2]$ **(VIII)** | 625 | 5000 | 2500 |
| $[ZnBr_2(Nia^+CH_2COO^-)_2]$ **(IX)** | 1250 | >5000 | 2500 |
| $[ZnI_2(Nia^+CH_2COO^-)_2]$ **(X)** | 1250 | 2500 | 5000 |
| $[Zn(ClH_2COO)_2(Caf)_2] \cdot 2H_2O$ **(XI)** | 1250 | 5000 | 5000 |
| $[Zn(BrCH_2COO)_2(Caf)_2] \cdot 2H_2O$ **(XII)** | 625 | 1250 | 2500 |
| $Zn(CH_3CH_2CH_2COO)_2$ **(XIII)** | 312 | 5000 | 5000 |
| $Zn((CH_3)_2CHCOO)_2$ **(XIV)** | 312 | 5000 | 5000 |

The efficiency of these four carboxylates against *Staphylococcus aureus* is good, but almost no inhibitory effect was observed on *Escherichia coli*. In the case of yeast *Candida albicans* there is evident lowering of efficiency from formiate to butyrate. Zinc formiate exhibits good efficiency, the biological activity of the butyrate and isobutyrate is very low. No effect was observed in connection with the branching of the carboxylate chain (**XIII, XIV**).

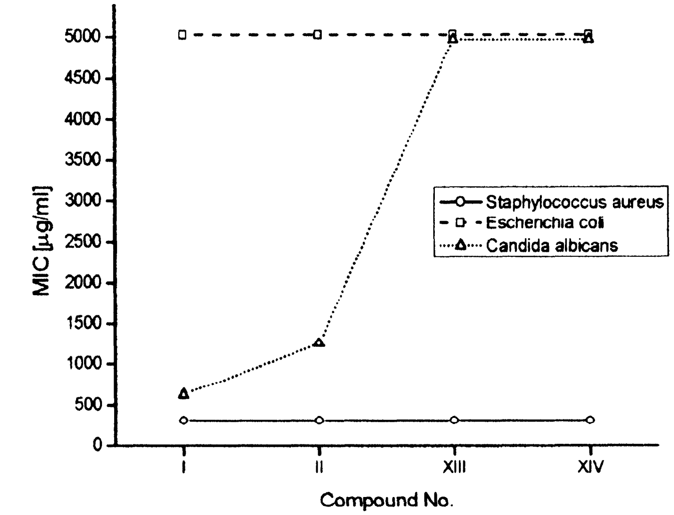

Fig. 2. The influence of the carboxylate chain length on the biological activity

The influence of the substitution of the hydrogen atom of the zinc(II) acetate by halogen is illustrated in Fig. 3. The presence of halogen atom in the molecule can affect the biological activity of the compound by steric or electron effects. As it is obvious from Fig. 3 substitution of the hydrogen atom has no or minimal effect for yeast *Candida albicans*. In the case of both bacterial strains the efficiency increases in the order of H < Cl < Br < I. From overall point of view the compounds $Zn(XCH_2COO)_2 \cdot nH_2O$ (X=Cl, Br, I; n=0, 2) have the best activity against *Staphylococcus aureus*, medium activity against *Candida albicans* and their activity in the case of *Escherichia coli* rises in the order $Zn(CH_3COO)_2 \cdot 2H_2O$ < $Zn(ClCH_2COO)_2 \cdot 2H_2O$ < $Zn(BrCH_2COO)_2 \cdot 2H_2O$ < $Zn(ICH_2COO)_2$. The similar results were observed in the study of copper carboxylates, where efficiency of the compounds linearly increases with the substituting halogen F < Cl < Br < I [25].

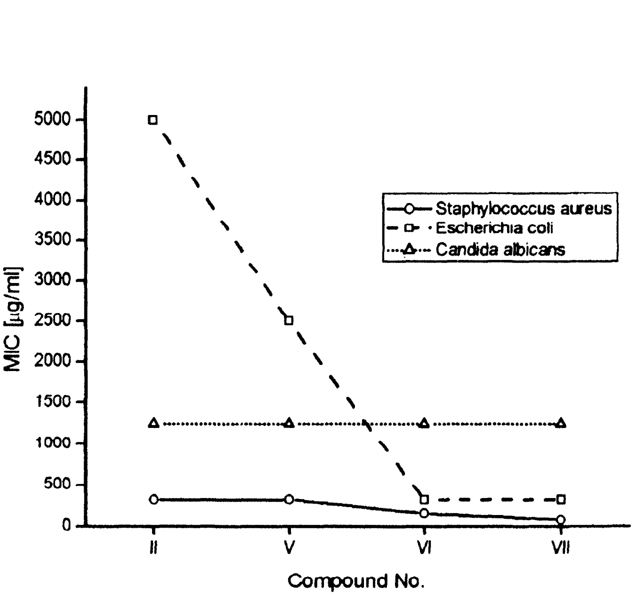

Fig. 3. The influence of the halogen atom on the biological activity of zinc(II) carboxylates

The organic ligands can contribute to the better transport of metal ions through the lipophillic regions of cell membranes. The influence of ligands nicotinamide and caffeine on the biological activity is shown in Fig. 4. Nicotinamide and caffeine complexes did not exhibit any higher efficiency compared with the simple zinc(II) carboxylates and mostly their efficiency even decrease. The same negative effect of the organic molecule on the efficiency of carboxylates was observed by Melník et al who studied copper carboxylates with antipyrine [25].

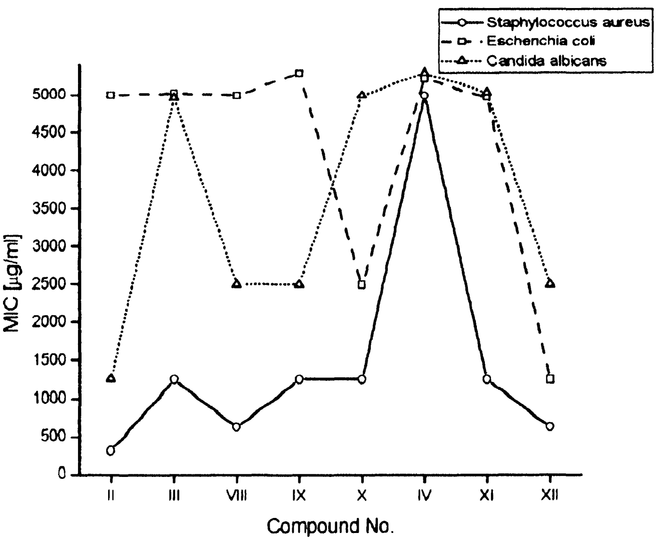

Fig. 4. The influence of the ligands on the biological activity of zinc(II) carboxylates

The nicotinamide complexes are more effective than the caffeine complexes. The efficiency of the complexes decreases in the order *Staphylococcus aureus* > *Candida albicans* > *Escherichia coli*. The positive effect of the halogen atom on the efficiency of the complexes, described already above, can be observed again in Fig. 4.

In conclusion, no correlation can be made between assigned structural types (see Fig. 1) and biological activity. This is probably due to the low thermodynamic stability of the prepared compounds, their dissociation in the solution and consequently lower permeability through cell membranes.

**Acknowledgment**

This project was supported by Slovak grant agency VEGA, grants no. 1/9247/02 and 1/7202/20, respectively. This financial support is gratefully acknowledged.

**References**

1. B. L. Vallee, D. S. Auld: *Biochemistry*, **24**, 5647(1990)
2. E. Kimura, T. Koike: *Advances in Inorganic Chemistry*, **44**, 229(1997)
3. W. Kaim, B. Schwederski: *Bioinorganic Chemistry: Inorganic Elements in the Chemistry of Life*, John Wiley & Sons, Chichester, England, 1994
4. S. C. Cunnane: *Zinc: Clinical and Biochemical Significance*, CRC Press, Boca Raton, Florida, USA, 1988
5. B. D. Korant, J. C. Kauer, B. F. Butterworth, *Nature*, **248**, 588(1974)
6. E. De Clercq: *Metal-Based Drugs*, **4**, 173(1997)
7. C. L. Feucht, B. S. Allen, D. K. Chalker: *J. Am. Acad. Dermatol.*, **3**, 483(1980)
8. R. B. Martin: *in Metal Ions in Biological Systems*, Vol. 20, H. Sigel, Ed. Marcel Dekker, New York and Basel, 1986
9. Z. H. Chohan, S. K. A Sherazi: *Metal-Based Drugs*, **4**, 327(1997)
10. K. Györyová, V. Balek: *J. Therm. Anal.*, **40**, 519(1993)
11. V. Zelenák, K. Györyová, J. Loub: *Main Group Met. Chem.*, **18**, 211(1995)
12. K. Györyová, V. Zelenák, V Balek: *Thermochim. Acta*, **234**, 221(1994)
13. V. Zelenák, K. Györyová, J. Simon: *J. Therm. Anal.*, **46**, 573(1996)
14. V. Zelenák, K. Györyová, J. Loub, I. Císarová: *Acta Cryst.*, **C52**, 1917(1996)
15. V. Zelenák, K. Györyová, J. Loub, I. Císarová: *Acta Cryst.*, **C52**, 808(1996)
16. V. Zelenák, K. Györyová: *Proc. 15th Conference on Coordination Chemistry*, Smolenice, Slovakia, 1995, p. 167-172
17. K. Györyová, J. Kovarová, E. Andogová, V. Zelenák, F. A. Nour El-Dien: *J. Therm. Anal. Calorim.* **67**, 119(2002)
18. V. Zelenák: Unpublished research
19. M. K. Johnson, D. B. Powell, R. D. Cannon: *Spectorchim. Acta*, **37A**, 899(1981)
20. T. Ishioka, A. Murata, Y. Kitagawa, K. T. Nakamura: *Acta Cryst.*, **C53**, 1029(1997)
21. W. Clegg, D. R. Harbron, Ch. D. Homan, P. A. Hunt, I. R. Little, B. P. Straughan: *Inorg. Chim. Acta*, **186**, 51(1991)
22. W. Clegg, I. R. Little, B. P. Straughan: *Acta Cryst.*, **C42**, 1701(1986)
23. Von. N. Burger, H. Fuess: *Z. Kristallogr.*, **145**, 346(1977)
24. J. Blair, R. A. Howie, J. L. Wardell: *Acta Cryst.*, **C49**, 219(1993)
25. M. Melník, A. Auderová, M. Holko: *Inorg. Chim. Acta*, **67**, 117(1982)

**Received: September 11, 2000 – Accepted: January 23, 2001 – Accepted in publishable format: March 19, 2002**

